# Cancer invasion and anaerobic bacteria: new insights into mechanisms

**DOI:** 10.1099/jmm.0.001817

**Published:** 2024-03-28

**Authors:** Rachel Hurst, Daniel S. Brewer, Abraham Gihawi, John Wain, Colin S. Cooper

**Affiliations:** 1 Norwich Medical School, University of East Anglia, Norwich, Norfolk, NR4 7TJ, UK; 2 Earlham Institute, Norwich Research Park Innovation Centre, Colney Lane, Norwich NR4 7UZ, UK; 3 Quadram Institute Biosciences, Colney Lane, Norwich, Norfolk, NR4 7UQ, UK

**Keywords:** aggressive cancer, anaerobic bacteria, bacterial invasion, cancer, infection, intracellular bacteria, mechanisms, metastases, pathogenic, polymicrobial

## Abstract

There is growing evidence that altered microbiota abundance of a range of specific anaerobic bacteria are associated with cancer, including *Peptoniphilus* spp., *Porphyromonas* spp., *Fusobacterium* spp., *Fenollaria* spp., *Prevotella* spp., *Sneathia* spp*., Veillonella* spp*.* and *Anaerococcus* spp. linked to multiple cancer types. In this review we explore these pathogenic associations. The mechanisms by which bacteria are known or predicted to interact with human cells are reviewed and we present an overview of the interlinked mechanisms and hypotheses of how multiple intracellular anaerobic bacterial pathogens may act together to cause host cell and tissue microenvironment changes associated with carcinogenesis and cancer cell invasion. These include combined effects on changes in cell signalling, DNA damage, cellular metabolism and immune evasion. Strategies for early detection and eradication of anaerobic cancer-associated bacterial pathogens that may prevent cancer progression are proposed.

## Infectious agents and anaerobic bacteria associated with cancer

There are currently ten well-established carcinogenic infectious agents associated with cancer development, including *Helicobacter pylori* causing stomach cancer, hepatitis B and C virus causing liver cancer, human papilloma virus causing cervix uteri cancer and *Schistosoma haematobium* causing bladder cancer [1], producing an estimated 2 million cancer cases each year [1]. There is growing evidence to indicate that many other pathogenic and clinically significant bacterial species may also play a role in cancer development and/or progression at multiple cancer sites, perhaps most strikingly for specific anaerobic bacteria as detailed in Table 1 (see also Table S1, available in the online version of this article). These are the topic of this review. Specifically, we present new observations and hypotheses involving interlinked mechanisms of polymicrobial infections (complex mixtures of multiple) anaerobic bacteria and cancer, focusing on how multiple anaerobic bacteria may work together to promote cancer development and cancer progression.

**Table 1.** Anaerobic bacteria taxa (phyla, genera and species), identified in clinical samples reported to be associated with cancer

Bacteria phyla	Bacteria genera	Bacterial species	Association with cancer type	Summary cancer types
*Actinobacteria / Actinomycetota*	*Propionimicrobium*	*P. lymphophilum*	*P. lymphophilum* more abundant in urine samples from prostate cancer patients compared with controls [66].	Prostate
	*Varibaculum*	*V. cambriense*, *Varibaculum* sp. nov./ *Varibaculum prostatecancerukia*	*V. cambriense* more abundant in urine samples from patients with prostate cancer compared with controls [66,146]. *Varibaculum* one of several taxa enriched in urine samples from bladder cancer cases compared with controls [147].	ProstateBladder
*Bacteroidetes / Bacteroidota*	*Prevotella*	*P. timonensis*, *P. bivia P. buccalis*, *P. corporis*, *P. disiens*, *P. melaninogenica, P. nigrescens, P. pallens, P. nanceiensis, P. copri*	Altered abundance of *Prevotella* sp. with other microbiota associated with laryngeal squamous cell carcinoma [148], gingival and oral squamous cell carcinomas [149,151], intestinal metaplasia [152], colorectal cancer [153,157], breast [158], cervical, endometrial [159], gastric [128,160,162], lung [163, 164] and bladder cancer [165]. *Prevotella* species associated with cancer include: *P. copri* plus other bacteria associated with gastric cancer [161,166], *P. bivia* and cervical cancer [167,168], *P. timonensis* and high-grade cervical intraepithelial lesions [130]. *P. melaninogenica*, *P. pallens*, *P. nigrescens*, *P. nanceiensis* associated with oral squamous cell carcinoma [169]. *P. buccalis* one of several anaerobes detected in melanoma metastases [170]. *P. nanceiensis* associated with higher risk oesophageal squamous cell carcinoma [171].	LaryngealOralGingivalColorectalBreastCervicalEndometrialBladderGastricLungMelanomaOesophageal
	Porphyromonas*	*P. bennonis*, *P. somerae*, *P. asaccharolytica, P. gingivalis, Porphyromonas* sp. nov.*/ Porphyromonas bobii*, *P. uenonis, P. endodontalis*	Several *Porphyromonas* sp. [67] including *P. asaccharolytica* [172] and other anaerobic bacteria including *Fusobacterium* and *Prevotella* species detected with increased abundance in gut and cancer tissue associated with colorectal cancer [67,172]. Associated with high- grade/advanced prostate cancer in combination with other anaerobes [10], see Fig. 1. *P. gingivalis* associated with oral squamous cell carcinoma [173], higher risk oesophageal squamous cell carcinoma [137,171] and pancreatic cancer [174]. Novel uncultured *Porphyromonas* sp. together with other bacteria species associated with the presence of endometrial cancer [175]. *P. uenonis* one of several bacteria associated with cervical cancer [130]. *P. bennonis* one of several anaerobes detected in melanoma metastases [170]. Higher abundance of *P. endodontalis* associated with oral squamous cell carcinoma [169].	ColorectalProstateOralOesophagealPancreaticEndometrialCervicalMelanoma
	*Bacteroides*	*B. coagulansB. ovatusB. uniformisB. fragilisB. vulgatusB. dorei*	*Bacteroides* sp. isolates frequently isolated from clinical samples including cancer patients [176,177]. *Bacteroides* sp. and *Streptococcus* sp. enriched in rectal swab samples of prostate cancer patients compared with non-cancer controls [90]. *B. ovatus* and *B. uniformis* two of several anaerobes associated with increased risk of colorectal cancer diagnosis [178]. *B. fragilis* and *Prevotella* increased in abundance in faecal samples from colorectal cancer patients compared with control patients [157]. *B. vulgatus*, *B. ovatus*, *B. dorei* detected as intratumoural bacteria in melanoma metastases [170]. *Bacteroides* spp. and other faecal microbiota taxa associated with pancreatic ductal adenocarcinoma [179]. *Bacteroides* and other taxa enriched in gastric cancer tissue compared with control tissue [162] and one of several dominant genera in colorectal cancer [76].	Multiple cancer typesProstateColorectalMelanomaPancreaticGastric
*Firmicutes*/ *Bacillota*	Fenollaria* */ Ezakiella/ [Sporobacterium]*	*F. sporofastidiosus*	*Fenollaria/F. sporofastidiosus* associated with high-grade prostate cancer in combination with other anaerobes [10], see Fig. 1. ‘[*Sporobacterium*]’ decreased together with other microbiota in stool samples from colorectal cancer patients after curative surgery [180]. *Ezakiella* was identified as one of several anaerobic taxa enriched in vaginal microbiome of patients with cervical or endometrial cancer [158]. Also, *Ezakiella* one of several anaerobic bacteria genera detected in relatively high abundance in urine of male and female bladder cancer patients [165].	ProstateColorectalCervicalEndometrialBladder
	Peptoniphilus*	*P. harei*, *P. coxii*, *Peptoniphilus* sp. nov./ *Peptoniphilus rachelemmaiella*	*Peptoniphilus* spp. associated with high-grade advanced prostate cancer in combination with other anaerobes [10], see Fig. 1. Involved in polymicrobial infections, enriched abundance associated with endometrial cancer [175]. *P. harei* infection in lymphocele in prostate cancer patient post-prostatectomy [181] and one of several anaerobes frequently isolated from clinical samples from cancer patients [176]. *Peptoniphilus* together with other bacteria enriched in breast cancer tissue compared to healthy control tissue samples [158], also detected in relatively high abundance in urine of bladder cancer patients [165]. *Peptoniphilus,* and other anaerobic genera dominant in oral squamous cell carcinoma [76]. *Peptoniphilus* enriched in vaginal microbiota of endometrial and cervical cancer patients compared to controls with no cancer [159].	ProstateEndometrialBreastBladderOralCervical
	Anaerococcus*	*A. lactolyticus*, *A. prevotii, A. tetradius*	*Anaerococcus* sp. associated with high-grade advanced prostate cancer in combination with other anaerobes [10] see Fig. 1. *A. lactolyticus* more abundant in urine samples from prostate cancer patients compared with controls [66] *Anaerococcus* sp. and other anaerobes associated with high-grade cervical cancer [131] and enriched in vaginal microbiome of cervical and endometrial cancer patients compared to control group [159]. *Anaerococcus* one of several anaerobic bacteria genera in relatively high abundance in urine of bladder cancer patients [165] and identified as related to progression of cervical intraepithelial neoplasia [130]. *Anaerococcus* sp. and *Fusobacterium* sp. were two of several urinary microbiome bacteria associated with bladder cancer [182].	ProstateCervicalEndometrialBladder
	*Veillonella*	*V. atypicaV. parvulaV. dispar*	Microbiota in lower airway, including *Veillonella,* predictive for lung cancer [163] and lung cancer progression [138]. *Veillonella* was one of several anaerobic bacteria genera in relatively high abundance in urine of bladder cancer patients compared to controls [147,165]. *Veillonella atypica, Veillonella* spp. and other taxa enriched in faeces from patients with pancreatic ductal adenocarcinoma [179]. *V. dispar* and *V. parvula* detected in melanoma metastases [170]. Gastrointestinal microbiota including *Veillonella* significantly increased in acute lymphoblastic leukaemia in children compared with controls [183]. *Veillonella* detected in cancer tissue samples was one of eight taxa with altered abundance associated with gastric cancer [128].	LungBladderPancreaticMelanomaAcute lymphoblastic leukaemiaGastric
*Fusobacteria* / *Fusobacteriota*	Fusobacterium*	*F. nucleatumF. hwasookii*	*F. nucleatum* together with other bacteria associated with colorectal cancer [13,14, 178, 184,187], cervical cancer [188], prostate cancer [10] see Fig. 1. Enriched in breast cancer tissue [158,189]. *Fusobacterium* one of several dominant genera in colorectal cancer and oral squamous cell carcinoma [76]. *Fusobacterium* sp. with other microbiota in altered abundance associated with laryngeal and oral squamous cell carcinoma [148,190, 191], bladder cancer [182], gastric [128,162], plus cervical and endometrial cancer [130,159]. *Fusobacterium* and several other anaerobes including *Prevotella* and *Porphyromonas* in increased abundance in oral squamous cell carcinoma tumour compared with control healthy mucosa [150,151]. *F. nucleatum, F. hwasookii, A. omnicolens, Veillonella atypica, Veillonella* spp*. and Bacteroides* spp. enriched in faeces from patients with pancreatic ductal adenocarcinoma [179]. *F. nucleatum* and other bacteria associated with breast and pancreatic cancer [12] and detected in melanoma metastases [170]. *F. nucleatum* enrichment in colorectal tissue associated with poor prognosis colon cancer [192]. In a meta-analysis of several studies *Fusobacterium* plus several other genera in increased abundance in gastric cancer tissue associated with gastric cancer progression [128].	ColorectalCervicalEndometrialProstateBreastLaryngealOralBladderMelanomaGastricPancreatic
	*Sneathia*	*S. sanguinegens*	Increased abundance of *Sneathia sanguinegens* and other anaerobes (*Anaerococcus* sp.) associated with high-grade cervical cancer [131]. Increased abundance of *Sneathia* and other anaerobes associated with cervical and endometrial cancer [130,159].	CervicalEndometrial
Multiple anaerobes including those belonging to phyla *Bacteroidetes, Firmicutes and Fusobacteria*	Multiple genera including Porphyromonas*, *Prevotella*, Bacteroides,Veillonella, Fenollaria*, Ezakiella/[Sporobacterium], Peptoniphilus*, Anaerococcus*Fusobacterium*Sneathia	Multiple species, polymicrobial infection	See above, most studies [10,66, 67, 76, 128, 130, 147, 148, 150, 151, 158,160, 162, 169] report altered abundance of several anaerobes, often co-occurring as polymicrobial infection, that are associated with multiple cancer types. Increased number of anaerobes cultured from prostate cancer tissue compared with benign tissue (bacteria not fully characterized) [17]. Multiple bacteria in tissue, including *Fusobacterium*, associated with many types of cancer [11], analyses of data [11] indicated other anaerobes present in cancer tissue. Intracellular bacteria associated with multiple cancers including ovary, lung, bone, colorectal, breast, taxa including anaerobe *Bacteroidia*, *Prevotellaceae*, in addition, *Fusobacterium* sp. associated with breast and pancreatic cancer [12]. *Clostridia* class of bacteria and *Bacteroidia* two anaerobic taxa enriched in pancreatic cancer tissue associated with pancreatic cancer patient shorter term survival [129].	ProstateColorectalCervicalBreastEndometrialLaryngealOralGastricPancreaticBladderLungOesophagealMelanoma

♦*Anaerobic bacteria biomarkers set (ABBS) associated with prostate cancer progression and also associated with several other types of cancer as detailed above.

**Fig. 1.**
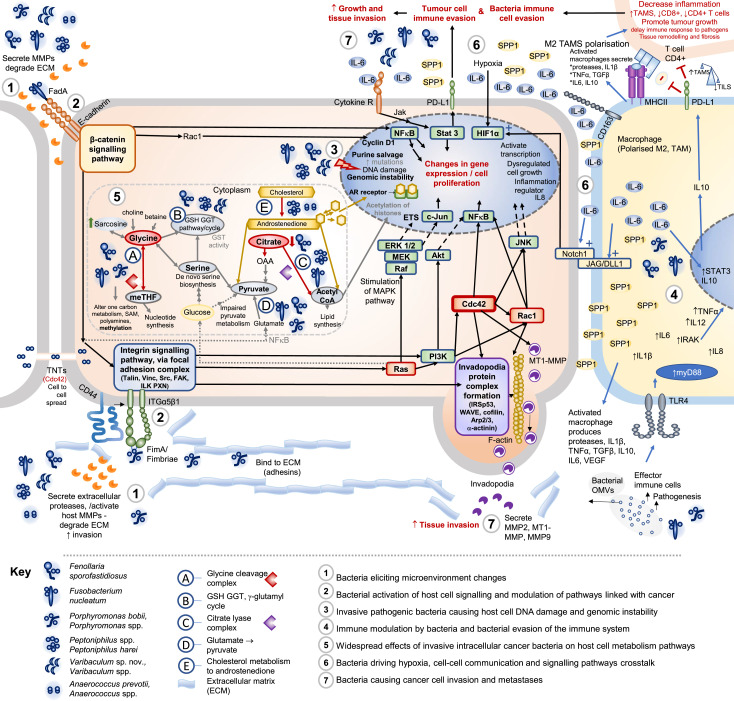
The seven steps for how human cell invasive pathogenic bacterial species may cause cancer and invasion. The key bacteria involved are included in the legend, other bacteria may be involved at specific steps as discussed in the text and detailed in Table 1, including *Sneathia* spp., *Prevotella* spp. and *Veillonella* spp. Early events of effects of cancer associated bacteria steps 1 to 3, proposed later events of cancer-associated bacteria steps 4 to 7 (also including step 2). The detailed mechanisms of action of the anaerobic bacteria causing host cell and tissue changes associated with carcinogenesis and cancer cell invasion and the proposed order of events are described, several of the mechanisms in the steps are strongly supported by evidence and some are hypotheses, as described in further detail below in the review main text, steps 1 to 7.

Other infectious agents and their links to cancer have been reviewed elsewhere [2,8] including *Mycoplasma* spp. [3,4], microaerophilic *Campylobacter* sp. [5], facultative *Salmonella* [8] and microaerophilic/capnophilic *H. pylori* [2]; where relevant we mention the known oncomicrobe *H. pylori* for comparison. Associations of bacteria to anticancer drug response are also presented elsewhere [9].

Recently, data have linked specific pathogenic anaerobic bacteria to aggressive disease. The bacteria *Anaerococcus* spp*., Fusobacterium* spp*., Peptoniphilus* spp*., Porphyromonas* spp. and *Fenollaria* spp. called the Anaerobic Bacteria Biomarkers Set (ABBS) [10] are associated with high-grade prostate cancer, cancer progression and increased risk of metastases. Notably, many of these bacteria are also associated with other cancer types (Tables 1 and S1). Thus, specific bacteria can potentially have roles in the development of multiple cancers, contrasting with the recent view that bacteria may be specific to and used in the diagnosis of individual cancer types [11]. Cancer types associated with anaerobic pathogenic bacteria and/or microbiota dysbiosis additionally include prostate, oral/head and neck, oesophageal, gastric, pancreatic, colorectal, breast, endometrial, cervical, acute lymphoblastic leukaemia, melanoma, bladder and lung cancer (detailed in Tables 1 and S1). In our own analysis of public datasets (unpublished), we found evidence that ABBS bacteria were present in cancer tissues from stomach, colorectal, oesophageal, head and neck, breast, cervical, bladder, and prostate cancer in agreement with Table 1, but also in ovarian, and kidney cancer.

Importantly bacteria associated with cancer are commonly present intracellularly, inside the human cells [10,12], and invasive within cancer tissue [12,15]. The terms used to describe the bacteria were ‘intracellular bacterial pathogens’ and ‘intratumour(al) bacteria’ [12,13, 15]. A review of specific cancer-associated bacteria and the mechanisms used to invade and persist within human host cells is included below. *H. pylori* a known carcinogenic bacterial pathogen, can also be invasive and intracellular (reviewed in [16]). Publications from the 1970s and 1980s [17,18] link anaerobic bacteria and cancer but the exact identities of the bacteria could not, at the time, be easily determined [17,18]. Recent technologies have allowed the identification of specific bacteria associated with cancer types (Tables 1, and S1) leading, through experimentation and genome analysis, to hypotheses and discoveries of how the bacteria, including recently isolated species (Table 1), may contribute to cancer progression. We hypothesise that there may be common inter-linked mechanisms for the effect of invasive cancer-associated anaerobic bacterial pathogens on cancer progression relevant to several cancer types.

The proposed mechanisms of action of polymicrobial anaerobic bacteria combined to cause cancer development and cancer invasion are presented in steps 1 to 7 in this review. We provide a review of the seven steps of how multiple anaerobic bacteria may contribute to both the early events in cancer development steps 1 to 3 causing several changes in normal cells to cancer development. In addition also to the proposed later events of the effects and mechanisms of action of cancer-associated anaerobic bacteria discussed from steps 1 through 7 to cause cancer progression and invasion. The seven steps could be considered as hallmarks of cancer associated with anaerobic cancer pathogens.

### Step 1 Bacteria eliciting microenvironment changes

One of the early events of bacterial effects on the human host includes bacterial secretion of proteases, including metalloproteases, and activation of host cell matrix metalloproteases (MMPs), leading to degradation of the extracellular matrix (collagen, laminin, fibronectin). For reviews on the impact of bacterial pathogens and effects on host extracellular matrix in other models see [19] and [20]. Anaerobic cancer-associated bacteria proposed to be involved in this process (Fig. 1, step 1), include *F. sporofastidiosus*, *F. nucleatum*, *Porphyromonas* spp., *Varibaculum* spp., *Anaerococcus* spp. and *Peptoniphilus* spp. that encode predicted HtrA/DegQ family proteins, FtsH metalloprotease zincins, PrsW protease, serine protease and/or predicted collagenase activity [10]. Collagenase activity, predicted in *F. sporofastidiosus*, *Porphyromonas* spp., *Peptoniphilus* spp. and *Anaerococcus* spp., could enable the bacteria to cause extracellular matrix remodelling. In addition, bacterial secreted HtrA can open cell-to-cell junctions and break down the epithelial barrier [21,23], allowing bacteria to access the basement membrane. This would in turn enable bacterial pathogen interaction with integrins leading to subsequent signalling pathways plus E-cadherin-β-catenin signalling, see step 2. Anaerobic bacteria can also decrease pH in the microenvironment, with the low pH leading to the high affinity extended open state integrin headpiece [24] allowing bacterial binding, integrin activation and signalling mechanisms, as outlined in step 2A.

**Mechanisms Step 1**: multiple anaerobic cancer-associated bacteria cause altered MMPs proteases, E-cadherin cleavage, allowing the bacteria to access the basement membrane and cause other microenvironment changes in human host tissue associated with cancer.

Several of the cancer anaerobic bacteria effects on the microenvironment have also been associated with later stages of cancer progression and invasion. *F. nucleatum* and *Prevotella* sp. have been documented to increase secretion of host MMPs, including MMP7, MMP13, MMP9, activated MMP9 [19,25], involved in degradation of extracellular matrix, contributing both to increased invasion of the bacteria into the tissue and to cancer invasion [26,27]. *Porphyromonas* spp. *P. bobii* and *Fusobacterium nucleatum* encode predicted components of the Tol-Pal system (including Tol R, Q, A, C, A, Ybg) and may secrete outer membrane vesicles (OMVs) and extracellular metalloproteases into the extracellular environment [20,28,30]. HtrA can also be secreted in bacterial OMVs [21] contributing to breakdown of epithelial barrier via cleavage of E-cadherin [21,23], enabling further bacterial invasion and microenvironment changes associated with cancer. Altered E-cadherin can have significant effects on cancer formation and progression [31,33]. Bacterial effects on the microenvironment, Mechanisms Step 1 (box 1), are also linked with later mechanisms, see steps 2 and 7, and Fig. 1.

### Step 2 Bacterial activation of host cell signalling events and modulation of pathways linked with cancer

#### Step 2A Bacteria interaction with integrin α5β1 and signalling pathway

Integrins are located on the basement membrane [34] and may be accessible to bacterial pathogen binding following changes in the microenvironment (step 1). Multiple bacterial species have been shown to interact with host cell integrins [20,35, 36], particularly species belonging to *Porphyromonas* genera [20] (Table 1). Bacteria may also bind to CD44 and/or ITGα5β1, other integrins such as αvβ3 and αvβ5 [20,35, 37], plus may interact with integrins directly or using vitronectin as a crosslinker between bacteria and host [20,35, 37].

Integrin binding and activation of host cell-signalling events is one mechanism by which bacteria can stimulate their own uptake and invasion into the human cell: for review articles see [20,35, 38]. The summary of steps involved in bacteria and integrin binding plus the subsequent activation of host cell signalling pathways is described below and is compiled from multiple sources [19,20, 35,42] to reach a consensus. What is striking is that these same cell signalling cascades and proteins involved in response to the bacteria are also frequently aberrant in multiple types of cancer [33,43] and thus lead to the Mechanisms Step 2 in box 2 below plus mechanisms detailed in Fig. 1.

Bacteria including *Porphyromonas* sp. bind to integrins via fimbriae, (e.g. FimA; other bacteria may use adhesins IpaA/Ipa complex, ApaH [20] or bind via vitronectin [35]), resulting in formation of focal adhesion complexes [20,35] and recruitment, within the human cell, of focal adhesion proteins, talin, vinculin, activated focal adhesion kinase (FAK), Src, paxillin (PXN), and integrin-linked kinase (ILK) [20,35, 37]. In turn this leads to activation of Ras, PI3K, GTPases Cdc42, Rho and Rac1 [20,35, 36, 38, 41] (See Fig. 1). Filopodia/invadopodia and membrane ruffle formation then ensues via invadopodia protein complex formation including α-actinin, Rac1 activation and formation of complex with IRSp53, WAVE complex, cofilin and the Arp2/3 complex at the site [20,35]. Cofilin and Arp2/3 are involved in F-actin polymerization [20]. Other proteins implicated as involved in bacterial invasion invadopodia complex, actin nucleation and polymerization (depending on cell type) include N-WASP (Cdc42 activates N-WASP), profilin, Nck and cortactin (cortactin and vinculin have F-actin binding sites) [20,35, 38, 39, 41, 43]. Cdc42 also regulates MMPs at the protruding tip of the invadopodia including membrane type 1 matrix metalloproteinase (MT1-MMP/MMP14), with subsequent extracellular matrix degradation and formation of filopodia/ invadopodium, membrane ruffles and bacteria uptake into cell [19,20, 35,43]. All of the proposed interconnected signalling steps are summarized in Fig. 1. Bacterial activation of host cell signalling and modulation of pathways is also associated with host cell changes in gene expression/cell proliferation (Fig. 1, step 2 summary). These bacterial effects on cell signalling and in particular on Cdc42 are also critical for later steps in cancer progression including cancer invasion (Fig. 1, step 7 [20,43]).

#### Step 2B Bacteria interaction with E-cadherin receptor and cell signalling events

In an established mechanism, *F. nucleatum* binds E-cadherin receptor via bacterial FadA adhesin stimulating activation of the host β-catenin signalling pathways and downstream effects on the human host cell [6,44]. Host changes include increased gene expression of NFKβ, IL8, IL6 and Wnt, alteration in oncogenes Myc and Cyclin D1, and Rac1 and Cyclin D1 activation, overall causing increased growth stimulation of the host cell and facilitating bacteria uptake [6,44, 45]. Additionally, there is cross talk between the E-cadherin-β-catenin signalling pathway and integrin pathway as illustrated by the combined effects on PI3K, Cdc42 and Rac1 activation [34]. Activation of the Wnt/β-catenin pathway in human cells results in nuclear accumulation of β-catenin and aberrant accumulation of β-catenin in the nucleus of cancer cells has frequently been reported [46]. Bacteria can also directly mediate nuclear accumulation of β-catenin, for example, via protease mediated interaction with E-cadherin, causing dissociation of β-catenin from E-cadherin allowing β-catenin to be translocated to the nucleus where the effects subsequently result in altered gene expression and cell proliferation [46,47].

Steps 2A and 2B above facilitate the uptake of bacteria into the host cell together with modulation of host signalling pathways, including activation of Ras signalling pathways and small GTPases Cdc42, Rac1, stimulation of MAPKs, JNK [20], PI3K, Akt, NFKβ [20,35, 36, 38, 41, 42]. In turn, JNK, Ras and MAPK, ERK are known ETS modulators [48] (Fig. 1) potentially inducing an aggressive/invasive phenotype in multiple cancer types [49]. The combined consequences of bacteria on the human host cell pathways include: (i) changes in human host cell gene expression; (ii) modulation of inflammation regulators including IL8; (iii) increased cell proliferation; (iv) suppression of apoptosis/cell death and (v) dysregulated cell growth [6,20, 34,39, 41, 42, 44] (Fig. 1).

Overall, the pathways of effects of bacteria upon host cell invasion and cell signalling (Mechanisms Step 2) are frequently detected as aberrant pathways in carcinogenesis [33,40, 43] therefore may indicate a potential polymicrobial pathogen bacterial driven process.

**Mechanisms Step 2**: multiple bacterial species interact with human cells facilitating bacterial uptake and cause aberrant cell signalling (incl. GTPases, Ras, MAPK, PI3K, Cdc42 pathways), promoting cancer progression.

### Step 3 Invasive pathogenic bacteria causing host cell DNA damage and genomic instability

Infected host cells are often unable to eradicate bacteria, which then grow intracellularly in the nutrient rich cytoplasm and may locate to the cells perinuclear region [20], close to the nuclear membrane [12]. There they are well placed to cause chromosome damage, including single strand and double-strand breaks, through secreting nucleases and impairing DNA repair, similar to the mechanisms observed for *H. pylori* [50,52]. In lung cancer the presence of *Porphyromonas*, *Fusobacterium* and *Bacteroides* genera were significantly associated with chromosomal aberrations [53]. *Fusobacterium* spp. are known to encode and secrete extracellular deoxyribonucleases [10,54], and elevated levels of *F. nucleatum* DNA in colorectal cancer tissue sections were (i) detected close to the marker γH2AX for DNA double-strand breaks, and (ii) found to be associated with increased number of somatic mutations and microsatellite instability [55,56].

Other bacterial infections have been associated with host cell DNA damage via known mechanisms, for example, specific strains of *E. coli*, may have pathogenicity island *pks* that encodes enzymes for synthesis of the DNA damaging agent colibactin [57,58]. *pks*^+^ *E.coli* are associated with human host cell DNA damage and oncogenic gene fusions in prostate cancer [58] and with an oncogenic mutational signature in colorectal cancer [59]. Known toxic bacterial metabolites including nitrosamines may also cause host cell DNA damage [2] and bile acid degradation products produced by bacteria can result in increased reactive oxygen and nitrogen species leading to increased DNA damage (reviewed in [2]).

With respect to the role of the anaerobic bacteria in step 3, several bacteria listed in Table 1 (*F. sporofastidiosus, Porphyromonas* sp*., P. bobii, Peptoniphilus* sp*., Peptoniphilus rachelemmaiella*, *Anaerococcus prevotii, Fusobacterium nucleatum*) encode predicted MutS homologs (MutS, MutS2) but not MutH (extended data ref [10]), and thus are similar to *H. pylori* in lacking the MutH protein required for the methyl-directed MutSLH DNA mismatch system [60]. Chronic persistent bacterial pathogen infection may then alter the human host cell mismatch repair system as does *H. pylori* [50,51, 60]. Several strains with homologs MutS and MutS2 (*F. sporofastidiosus*, *P. bobii*, *Peptoniphilus harei, Peptoniphilus rachelemmaiella, Anaerococcus prevotii*) also lack MutY and MutM, a genotype reported to be more common in clinical bacteria associated with long term infection persistence [61], and with enhanced G:C to T:A transversions [50,60,62]. *F. sporofastidiosus*, *P. bobii*, *F. nucleatum, Peptoniphilus harei* and *Peptoniphilus rachelemmaiella* have both MutS and RuvC endonuclease that support genetic DNA recombination to provide oxidative DNA damage repair in the bacterial cells [60] to the benefit of bacterial growth. *Varibaculum prostatecancerukia* identified in prostate tissue [10] encodes purine deoxyribonucleosides salvage pathway components, similar to *H. pylori* [52]*,* enzymes and extracellular nuclease capable of scavenging purine nucleotides from host DNA [52] causing DNA damage.

Overall the anaerobic bacteria associated with cancer are predicted to be involved in host cell DNA damage, increase in mutations, potential gene rearrangements/fusions and impaired DNA repair (another one of the features of cancer [50,51, 63,65]) and lead to Mechanisms Step 3 (box 3), Fig. 1. There are also additional effects of anaerobic bacteria on histone acetylation and epigenetic effects detailed in step 5.

**Mechanisms Step 3**: multiple anaerobic bacteria species cause host cell genome instability, increased DNA damage and impaired DNA repair contributing to carcinogenesis.

### Step 4 Immune modulation by bacteria and bacterial evasion of the immune system

Bacterial infection can lead to inflammation in the tissue microenvironment [44,66,68] and bacteria can be detected intracellularly in immune cells within cancers [12]. Certain Gram-negative bacteria, including *Porphyromonas* sp. and *F. nucleatum* (Table 1), interact with immune cells such as macrophages through the Toll-like receptor 4 (TLR4) via recognition of bacterial endotoxin/ lipopolysaccharide (LPS) leading to macrophage activation with increases in MyD88, IL1β, IL6, IRAK, IL12, IL8 TNFα, STAT3 and IL10 production [3,13, 20, 69, 70] (Fig. 1). Over time, chronic infection may ensue with multiple intracellular invasive bacterial species infecting macrophages in the tissue environment [12,68, 71] with the bacteria surviving and multiplying intracellularly [68,71] leading to bacterial immune cell evasion and increased polarization of macrophages from the M1 to the M2 tumour associated macrophages (TAMs) [69,71]. The consequence is increased cytokine release: activated macrophages produce proteases, secrete cytokines including IL1β, TNFα, TGFβ, IL10, and IL6, and secrete osteopontin/secreted phosphoprotein 1, SPP1 [70,72] (Fig. 1). Contrasting to the initial induction of inflammation (see above), these changes cascade to anti-inflammation effects (including reduction in T lymphocytes), tissue remodelling, fibrosis and angiogenesis [70,72] in turn promoting tumour growth and reducing immune response to pathogens [68,70].

*Fusobacterium* spp. are correlated with markers of immune cells including dendritic cells and TAMs in human colorectal cancer tissue [13]. The change in the balance between increased levels of TAMs and reduced tumour infiltrating lymphocytes (TILs) leads to immune cell evasion for the bacteria, increased growth and spread/invasion into tissue. This is due to the reduction in CD8^+^, CD3^+^ and CD4^+^ T cells, TILs, in the environment which would typically contribute to the main defences against intracellular bacterial pathogens [68], but are significantly decreased by the effects of the bacteria, thus perpetuating further bacteria growth and survival.

The overall bacterial effects result in chronic cancer-promoting inflammation, with presence of certain sub-types of macrophages (including M2 TAMs), myeloid-derived suppressor cells (MDSCs), dendritic cells and other types of T lymphocytes (regulatory T cells) in cancer tissue also associated with cancer and progression/metastases [73,75]. In human colorectal cancer *F. nucleatum* was associated with recruitment of myeloid cells to the infected tissue and promoted changes associated with invasion [76]. In mouse models, more rapid intestinal and colon tumorigenesis induced by *F. nucleatum* [13] resulted in increased MDSCs, including monocytic and granulocytic MDSCs, which have strong immune suppressive effects, in particular suppression of CD4^+^ T cells [13]. The overall effects on immune cells (↑MDSCs, ↑M2 TAMs, ↓CD8^+^ CD4^+^ T cells) provide tumour enhancing activity and contribute to cancer progression [13,68, 70]. Indeed, removal of bacterial pathogens using an antibiotic cocktail (vancomycin, neomycin, metronidazole, amphotericin) in a mouse model reduced the MDSCs and increased M1 macrophage cells, promoting activation of CD4^+^ and CD8^+^ T cells, and protecting against invasive pancreatic ductal adenocarcinoma [77,78]. In several mouse model studies, microbiota have been associated with modulation of the tumour immune response [13,77, 78].

Downstream effects of *F. nucleatum* infection are in some cases linked to IL6 secretion by host cells [13,44] and activation of macrophages [13,70, 71]. IL6 can impact on neighbouring tumour cells via the IL6 cytokine receptor, causing activation of JAK and STAT3 signalling [79,81] (Fig. 1) leading to changes in gene expression and cellular proliferation [79,80]. Alteration of the STAT3 pathway is one of the aberrant signalling pathways contributing to the hallmarks of cancer [33]. Importantly, bacterial effects linked in with the downstream effects on STAT3 activation may also increase the expression of programmed death-ligand 1 (PD-L1) (Fig. 1). The effect on STAT3 and increased PD-L1 has been shown in several cancer types [82,83], leading to T cell suppression and tumour cell immune evasion [82,83] plus further contribution to bacteria immune evasion. TNFα and IL10 secreted by activated macrophages (Fig. 1) can also increase cancer cell and TAM expression of PD-L1 [70,83]. Indeed, *F. nucleatum* infection of cultured macrophages increased expression of TNFα and PD-L1 [84]. Bacterial OMVs secreted by *Porphyromonas* sp. and *F. nucleatum* have also been linked with effects on immune cells and modulation of immune response to pathogens, increasing the pathogenic effects of bacteria and enabling immune cell evasion [28,85,87]. Microbiota may in addition affect anticancer immunotherapy treatment, including anti-PD-L1 therapy [78], and other immunotherapies reviewed in [88,89].

Overall, the effects of the intracellular anaerobic bacterial infection on immune cells and signalling pathways are predicted to be substantial, allow the bacteria to grow undetected and to perpetuate, impacting cancer progression, leading to Mechanisms Step 4 (box 4).

**Mechanisms Step 4:** anaerobic cancer-associated bacteria may cause polarization of macrophages to M2 TAMs, and increases in MDSCs, leading to downstream effects on cytokines and modulation of signalling pathways resulting in increased angiogenesis, bacteria and cancer cell immune evasion and cancer progression /invasion.

Mechanisms Step 4 provides a potential route for treatment options for the cancer-associated anaerobic bacteria associated with immunosuppressive effects (see section below ‘*Potential options for treatment of cancer associated with anaerobic bacterial pathogens*’).

### Step 5 Widespread effects of invasive intracellular bacteria on host cell metabolism pathways

**Fig. 2.**
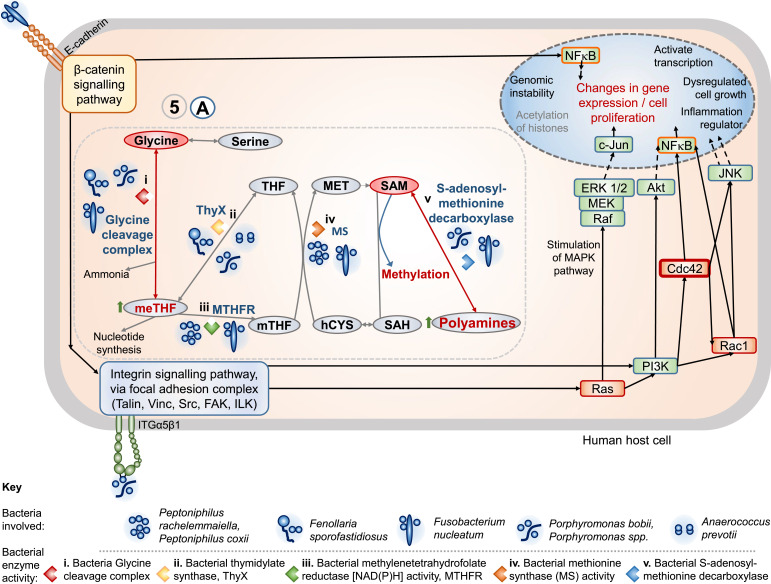
Step 5A, potential mechanisms of action of human cell invasive intracellular pathogenic bacterial species on host cell glycine metabolism and associated pathways. hCYS: homocysteine; MET: methionine; meTHF: 5,10-methylenetetrahydrofolate; MS: bacterial methionine synthase; mTHF: 5-methyltetrahydrofolate; MTHFR: bacterial methylenetetrahydrofolate reductase; SAH: S-adenosylhomocysteine; SAM: S-adenosylmethionine; THF: tetrahydrofolate; ThyX: bacterial thymidylate synthase. Impacts of specific bacteria on the host cell glycine metabolism and associated pathways are as indicated with the symbolized images of the bacteria displayed in the Fig. 2 key legend. Bacterial enzyme activities and enzyme complexes are detailed (i) to (v) above and in the review main text.

It is established that bacteria associated with cancer can perturb metabolism in host cells and tissue. This is illustrated by the gut microbiota modulation of short-chain fatty acids and downstream anti-inflammatory effects, altering host gene expression and cell proliferation [6]. Bacterial metabolites including nitrosamines and bile acid degradation products can result in host cell effects including increased DNA damage [2] (step 3). Gut microbiota enriched in patients with prostate cancer (including *Bacteroides*, see Tables 1, and S1) were associated with encoded folate, biotin and arginine metabolism pathways [90]. Microbiota have also been associated with metabolism of oestrogen the ‘estrobolome’ (reviewed in [91,92]). We have been able to postulate several interlinked effects of the intracellular anaerobic bacteria on host cell metabolism (Fig. 1, steps 5A to E and Fig. 2, step 5A with expanded details), relevant to several types of cancer as follows.

#### Step 5A Effects on glycine metabolism and associated pathways: folate, one-carbon and methylation

- Bacterial glycine cleavage complex – several of the anaerobic bacteria, *Porphyromonas* spp. (including *P. bobii*)*, Fusobacterium* spp. (including *F. nucleatum*) and *F. sporofastidiosus*, encode predicted bacterial multienzyme glycine cleavage complex [10] (including glycine decarboxylase/glycine dehydrogenase, glycine reductase, aminomethyltransferase). Via the glycine cleavage complex intracellular bacteria would metabolize glycine to ammonia, CO_2_, NADPH, with formation of 5,10-methylenetetrahydrofolate (meTHF); the latter used for nucleotide synthesis [93,94]. These alterations would modulate host cell glycine levels, increase meTHF (See Fig. 2, step 5A), and alter folate and one-carbon metabolism potentially impacting on DNA methylation. These pathways have also been identified as aberrant in cancer progression [95,97]. Increased activation of glycine and serine pathways and one-carbon metabolism have been linked to drivers of oncogenesis with associated epigenetic effects and cancer progression/development [95,97]. Increased glycine consumption in cancer is one of the main features identified in cancer metabolic reprogramming [95,96]. With flux regulation glycine can also be metabolized to form sarcosine, and choline and betaine can be metabolized to glycine (step 5, Fig. 1); this is relevant because glycine and sarcosine uptake and metabolism are linked to cancer invasiveness [96,98].
- Via bacterial thymidylate synthase – several of the anaerobic bacteria (*F. sporofastidiosus*, *Anaerococcus prevotii* and *Porphyromonas* spp.) also encode ThyX [10], thymidylate synthase, that catalyses meTHF to THF, for thymidylate synthesis, contributing to growth benefits and resistance to certain antifolate treatment [99] (with the bacterial glycine cleavage (i) described above affecting this metabolism via production of meTHF [94,99]), see Fig. 2.

Other features of the bacteria modulating host metabolite levels are via bacterial methylenetetrahydrofolate reductase [NAD(P)H] activity, (MTHFR) (iii) and via bacterial methionine synthase, (MS) (iv) see Fig. 2. Both MTHFR and MS are encoded in the genomes of anaerobic bacteria *Peptoniphilus* (including *P. rachelemmaiella* [10] and *P. coxii*) and *F. nucleatum*. The latter encodes a vitamin B12 dependent MS activation domain. Bacterial MTHFR activity catalyses the reaction meTHF to mTHF and MS catalyses the reaction of l-homocysteine and 5-methyltetrahydrofolate to methionine and tetrahydrofolate, with predicted impact on host cell metabolism (Fig. 2, steps 5A iii and iv).

Bacterial S-adenosylmethionine decarboxylase (v) (Fig. 2, 5A v) encoded by *Porphyromonas* sp. (including *P. bobii*) and *F. nucleatum* may impact on host S-adenosylmethionine (SAM), histone DNA and RNA methylation, methionine metabolism and polyamines [96,97, 100, 101]. An overview of the predicted effects of invasive intracellular bacteria on glycine and one-carbon host cell metabolism pathways are shown in Fig. 2.

In addition to the mechanisms shown in Fig. 2, changes in glycine metabolism may be linked to further downstream effects on *de novo* serine biosynthesis, impaired pyruvate metabolism, γ-glutamyl transferase (GGT) and glutathione (GSH) metabolism step 5B (see Fig. 1). Several of the anaerobic cancer-associated bacteria have relatively advanced antioxidant enzyme protection systems protecting against damage from the host cell and enabling growth of the bacteria intracellularly. For example, *F. sporofastidiosus* and *Peptoniphilus* sp. encode both protein–glutamine gamma glutamyl transferase (GGT) and glutathione-s-transferase (GST) (the latter also predicted in *Varibaculum* sp. nov.) [10]. Bacterial GGT and GST activities are quite rare [102,103], and provide protection against bacterial cell damage during growth and persistence of infection in human host cells [102,103]. Several of the anaerobic bacteria (*F. sporofastidiosus,* and *Peptoniphilus* sp.) also encode l-seryl tRNASec selenium transferase activity plus selenocysteine incorporation linked in with the importance of selenium in the glycine cleavage pathway in anaerobes (reviewed by Andreesen [94]).

Overall, the effects of the bacteria on glycine and one-carbon metabolism link in with other effects of other bacterial intracellular species on host cell citrate metabolism pathways and further alterations in metabolism pathways (Fig. 1, step 5).

#### Step 5C and D Effects on citrate metabolism

Several of the cancer-associated anaerobic bacteria: *Peptoniphilus* sp., (including *P. harei*), *F. sporofastidiosus* and *F. nucleatum,* encode a predicted citrate lyase complex [10] involved in degradation of citrate and in citrate metabolism pathways. Citrate is metabolized via the bacterial citrate lyase complex to oxaloacetate (that in turn may be converted to pyruvate), and acetyl CoA for increased lipid synthesis [93,104,106]. The bacterial ability to metabolize citrate from the surrounding environment, reviewed in [107], is quite a rare feature. Invasive intracellular bacterial species may alter host cell citrate metabolism for increased lipid synthesis and growth [104,105]. Notably aberrant citrate lyase activity is detected in many cancer types [106,108] with an increase in acetyl CoA and downstream metabolism associated with increased acetylation of histones affecting host global chromatin architecture and altering gene transcription [106,108, 109]. The effects of invasive intracellular bacteria on citrate metabolism together with the bacterial glycine cleavage complex (see above) may lead to epigenetic changes, DNA methylation and alteration of apoptosis [95,96, 106, 108] in the host cell.

This also links in with the effects of bacteria alterations on cell signalling pathways and oncoproteins (step 2B for example) with Ras activation, β-catenin and NFkB impacting on glutamate and glucose metabolism [110] (Fig. 1): glucose feeds into serine and glycine pathways, as well as pyruvate [96,108, 110]. In addition, *F. sporofastidiosus*, *F. nucleatum* and *Porphyromonas* sp. have predicted components for catalysing l-glutamate degradation VI to pyruvate step 5D (Fig. 1) [10], which combined with the other bacterial effects on pyruvate lead to altered pyruvate (Fig. 1).

#### Step 5E Cholesterol metabolism to androstenedione and androstenedione degradation

Several anaerobic bacteria (*Peptoniphilus* spp*.,* including *P. harei, P. rachelemmaiella*, *F. nucleatum* and *Anaerococcus prevotii*) are predicted to encode components of superpathways ‘cholesterol to androstenedione I (cholesterol oxidase)’ and ‘cholesterol to androstenedione II (cholesterol dehydrogenase)’ [10]. Only a few other bacterial species (including *Mycobacterium* sp*., Rhodococcus* sp. and *R. gnavus*) possess pathways needed for steroid synthesis [93,111, 112] and metabolites known to be produced by these few specific bacteria species include androstadienedione, testosterone and 1-dehydrotestosterone [111,114]. During bacterial cholesterol metabolism acetyl CoA is also produced [93,115] which may have potential epigenetic effects as described above in step 5C [106,108, 109] (Fig. 1). *Peptoniphilus* spp. (including *P. rachelemmaiella*, *P. coxii, P. harei*) and *Anaerococcus prevotii* also encode components of the androstenedione degradation pathway [10]. Steroid metabolism in bacteria [10,93, 111,113] has the potential to feed host steroid hormones production, impacting on hormone sensitive cancers (e.g. breast and prostate cancer) driving cancer progression and invasion [114,116, 117].

The wider contribution of metabolites from microbiota to the estrobolome (microbiota gene products that are capable of metabolizing oestrogens) is well established and reviewed in [92], as are its impact on oestrogen driven cancers, including endometrial and breast [92]. Metabolites may also impact on ovarian, cervical, gastric, prostate, bone and lung cancers [92,118]. Bacterial encoded enzymes are capable of producing oestrogen from cholesterol impacting on human host circulated and excreted oestrogen levels [92]. Several known anaerobes have this activity, including [92] *Clostridium perfringens*, *Bacteroides fragilis*, species belonging to *Firmicutes* (in particular *Clostridia* taxa), *Actinobacteria* and *Bacteroidetes* [118,119]. The activity is also predicted in anaerobic bacteria including *Varibaculum prostatecancerukia* and *Anaerococcus prevotii* [10].

Overall, the intracellular anaerobic cancer-associated bacteria have the potential to incur widespread effects on human host cell metabolic pathways (steps 5A to E, Figs1  2) relevant to cancer development and progression leading to Mechanisms Step 5 (box 5).

**Mechanisms Step 5**: intracellular anaerobic bacteria are driving the changes in human host cell metabolism (including glycine, citrate, cholesterol to androstenedione, anaerobic respiration) and methylation impacting through multiple mechanisms on cancer aggression and progression.

### Step 6 Bacteria driving hypoxia, cell-cell communication and signalling pathways crosstalk

There is support for a general link between the presence of bacteria and hypoxia. For example, *Mycobacterium tuberculosis* is associated with hypoxia in human tissue TB lesions [120]. *Fusobacterium* and *Porphyromonas* infected cell clusters in colorectal cancer, when compared with bacteria negative cell clusters, showed upregulation of pathways including those related to hypoxia [76]. In lung cancer, intratumoural bacterial infection burden was significantly correlated with *HIF1A* gene expression and hypoxia pathways [121]. HIF-1 is the key master regulator of hypoxia status and hypoxia-inducible factor 1-alpha (HIF-1α) has been shown to be overexpressed in many cancer types and is associated with cancer progression [122].

We propose that the anaerobic bacteria associated with cancer may induce hypoxia and promote cancer development. There are several observations that we are now drawing together that are consistent with this idea. Particularly several of the pathways expected to be activated by bacterial contact with human cells (step 2) have the potential to increase activity and/or stability of HIF-1α and contribute to cancer progression. These include Akt activation [110] (Fig. 1) and the Ras-MAPK pathways [27]. Additionally, SPP1 (also known as osteopontin/secreted phosphoprotein 1) [72,123] secreted by M2 macrophages (step 5) can modulate HIF-1α [70,123] (step 6, Fig. 1), and is associated with hypoxia and cancer metastases [123]. Secreted SPP1 may bind to CD44 and integrins [72,123] altering cell signalling pathways favouring cancer progression [123]. There are a number of additional complexities and supporting observations (see Fig. 1). Hypoxia and HIF-1α increase expression of integrins ITGA5, ITGβ1 [124], which could potentially create a positive feedback for enhancing further bacterial infection. In addition, the cellular effects of anaerobic bacterial infection on signalling pathways including, PI3K/Akt, NFKβ and Ras (step 2) may also impact on Notch signalling, Notch receptors and ligands, with direct crosstalk between signalling pathways [81,125]. Ras can activate Notch signalling and upregulate Notch ligands including Delta-like 1 (DLL1) [81]. Other cellular effects of bacteria on TGFβ, STAT3 and IL6, (step 4) may also upregulate/stimulate the Notch receptor and Notch ligands including JAG1 [81] and DLL1 [126] (see steps 4 and 6, Fig. 1) with engagement of Notch and Notch-ligand (JAG/DLL1) on neighbouring cell facilitating cell-cell communication and signalling [81,126, 127] (Fig. 1). This is relevant as Notch also has critical roles in hypoxia induced epithelial-mesenchymal transformation and cancer invasiveness [81,125]. In addition, Notch expression and ligands are found to be upregulated in many cancers including cervical, head and neck, renal, colon, pancreatic, breast and prostate [81,127].

These considerations lead to Mechanisms Step 6 (box 6).

**Mechanisms Step 6**: intracellular anaerobic bacteria are driving the changes in cell signalling causing hypoxia plus an increase in cell-to-cell communication and signalling pathway crosstalk increasing cancer progression.

### Step 7 Bacteria causing cancer cell invasion and metastases

**Fig. 3.**
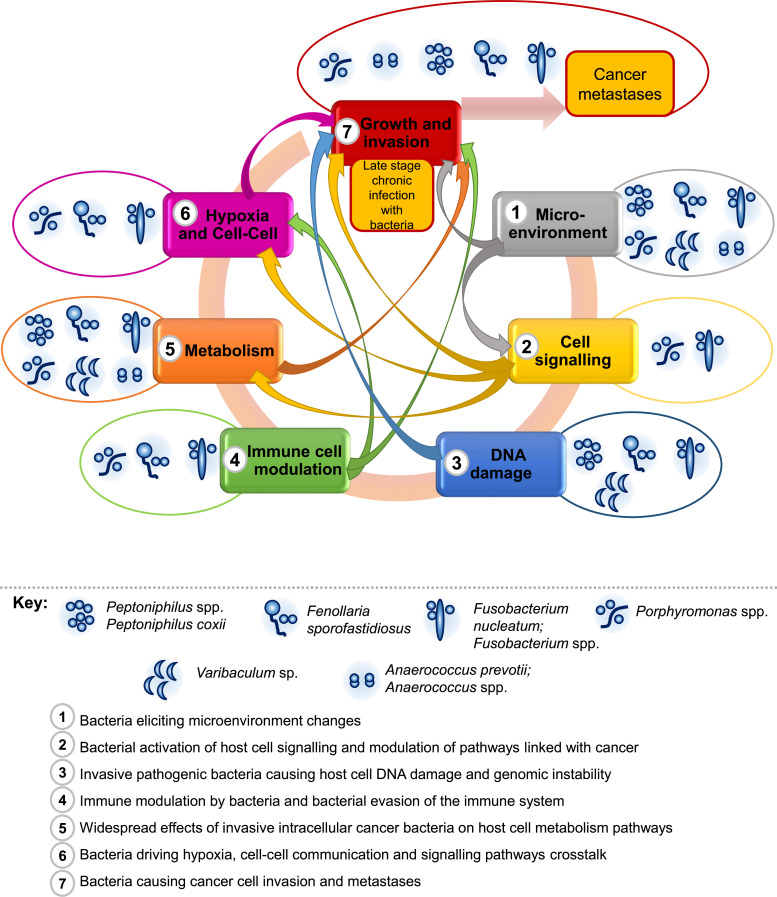
Summary overview of steps 1 to 7 and the interlinked mechanisms of how multiple invasive anaerobic bacteria may cause cancer and cancer progression. The key bacteria involved are included in the legend, other bacteria may be involved at specific steps as discussed in the text and detailed in Table 1, including *Sneathia* spp., *Prevotella* spp. and *Veillonella* spp.

We have proposed that mechanisms from steps 1 through 6 impact, either directly or indirectly, on step 7 leading to cancer invasion and metastases (Fig. 3).

Evidence for the association of anaerobic bacteria with cancer progression exists, including genera *Fusobacterium* and *Prevotella*, associated with gastric cancer progression [128], *Clostridia* and *Bacteroidia* associated with shorter survival time in pancreatic cancer [129], plus a group of five bacteria genera (*Fusobacterium*, *Anaerococcus*, *Fenollaria*, *Porphyromonas* and *Peptoniphilus*, ABBS) associated with prostate cancer progression [10]. Anaerobic bacteria associated with high grade cervical intraepithelial neoplasia were *Sneathia* sp., *Prevotella*, *Fusobacterium*, *Veillonella*, *Anaerococcus* and *Porphyromonas* species [130,131], with cervical cancer progression associated with higher levels of *Sneathia sanguinegens* [131] and *Anaerococcus* sp*.* [130].

Detailed mechanisms of how bacteria may be associated with cancer invasion are included in the steps above except for the following as a highlighted example. The effects of bacteria on cell signalling including Ras and Cdc42 [20,35, 36, 38, 41], MT1-MMP and invadopodia (detailed in step 2) associated with membrane ruffles and uptake of bacteria; incredibly similar pathways are detailed as associated with cancer invasion into the bloodstream and metastases [27,43, 132]. Cdc42 and MT1-MMP, increased by the downstream effects of intracellular pathogens on human host cells (see above steps), are critical for invasion of many cancer types [27] but also cdc42 may be linked with formation of tunnelling nanotubes which may enable further cell to cell spread of invasive intracellular bacteria (Fig. 1) [20,133,135]. Other detailed mechanisms are included in the steps above.

The mechanisms are interlinked as shown in Fig. 3 with several anaerobic bacteria (as listed in Table 1) associated with cancer progression in various types of cancer. The combined and interlinked effects of multiple invasive bacteria driving changes in cellular signalling pathways, gene expression changes, DNA damage, host cell metabolism, immune cell modulation and macrophage polarization, leading to widespread changes in human cells and the tumour microenvironment as described in the steps above (steps 1–6; Figs1  3). This may lead to promotion of cancer, increased growth and spread, increased tissue invasion, disruption of surrounding tissue structure and architecture, increased angiogenesis, and potential for increased cancer invasion to blood, nerves and lymphatic tissue. Known oncomicrobe *H. pylori* infection is significantly associated with advanced lymphatic metastases [136]. *Porphyromonas gingivalis* an anaerobic pathogen detected in oesophageal tissue was positively associated with lymph node metastases and poor overall survival rate in oesophageal squamous cell carcinoma patients [137]. In addition, airway microbiota enriched with *Veillonella parvula* in lung cancer patients was significantly associated with poor prognosis and cancer metastases [138]. Taken together these observations lead to Mechanisms Step 7, Fig. 1, summarised in box 7.

**Mechanisms Step 7**: long-term chronic infection with intracellular anaerobic bacteria in human host cells and cancer tissue drives cellular changes linked with cancer invasion, with interlinked ‘vicious circle’ effects of multiple invasive pathogenic bacteria leading to cancer progression and metastases.

## Potential options for treatment of cancer associated with anaerobic bacterial pathogens

The framework of mechanisms of action by which specific anaerobic bacteria may cause cancer (Fig. 1) provides treatment options to halt cancer development and progression. The aim would be to detect and eradicate anaerobic pathogens associated with cancer (Tables 1 and S1) to treat the cancer at the ‘top of the chain’, eradicate the anaerobic invasive pathogens and prevent effects of the intracellular pathogens on cancer progression.

Specific treatment options to eradicate the anaerobic invasive bacteria include modulation of macrophage polarization, specific targeted antimicrobial therapy and if late chronic stage infection in cancer tissue surgery to reduce high burden infected cancer tissue. The association of the anaerobic bacteria with immunosuppressive effects, particularly on polarization of macrophage to M2 TAMs (as discussed in step 4) presents options for immune modulation to increase success for the removal of the anaerobic bacteria. It is possible to reverse macrophage polarization of M2 TAMs back to M1 phenotype [70,71] resulting in recruitment of T cells into the tissue. Reversal of M2 to M1 macrophage may include via IFNα or CD40 activation [70], or via bacterial eradication – good antimicrobial options for emerging anaerobes include ertapenem, imipenem, meropenem, metronidazole, tigecycline, plus other antimicrobials [139]. An additional provision of immune modulation to increase healthy active CD4^+^, total CD3^+^, cytotoxic CD8^+^ T cells to the tissue to kill the remaining intracellular pathogens and cancer cells, based on mechanism of effects of the bacteria, would prevent cancer development and cancer progression.

Other routes of treatment for bacterial infection in cancer in the future may include phage-based therapy and engineered bacteria with care required for use of antibiotics as reviewed in [140].

We are proposing, based on steps 1–7 and the overarching mechanisms of action of the anaerobic bacteria linked to many cancer types as discussed in this review, that specific targeted antimicrobial therapy to remove the specific anaerobic bacteria (+/- surgery/immune therapy in chronic cases) would be an option for the prevention of cancer progression and metastases. A similar approach has been used for the oncomicrobe *H. pylori* with antimicrobial treatment options, protecting against *H. pylori* infection and gastric cancer (reviewed in [2,141, 142]). Eradication therapy to target *H. pylori* infection significantly reduced risk of gastric adenocarcinoma [143] and reduced cancer mortality [141,142]. Doxycycline antimicrobial treatment of infection in ophthalmic MALT lymphoma [144] and antimicrobial clarithromycin in advanced non-small cell lung cancer improved survival in patients with advanced non-small cell lung cancer [145]. There are a number of critical issues to consider when considering if antimicrobial treatment is appropriate. First, are all associations of bacteria with cancer development causal or is there a common underlying reason for observing an association of the presence of specific bacteria and aggressive disease? For example, a defect in the immune system promoting both cancer and bacterial growth – mechanisms that underpin each of the steps 1 to 7 would argue for the former. Secondly, where multiple bacteria are working together can a targeted treatment regime be developed that will penetrate the target tissue and eradicate the target anaerobic pathogens while sparing the commensal bacteria species.

## Concluding remarks and future perspectives

We have presented hypotheses of how multiple anaerobic pathogens may act together to cause cancer development and progression providing potential mechanisms detailed in the above steps 1 to 7 and summarized in Figs1^3^. Cancer types associated with anaerobic pathogenic bacteria include oral/head and neck, oesophageal, gastric, pancreatic, colorectal, breast, ovarian, endometrial, cervical, prostate, bladder, kidney, acute lymphoblastic leukaemia, bone, melanoma and lung cancer. Mechanistically, the anaerobic cancer-associated bacteria possess highly specialized homologs, unique bacterial proteins and machinery to alter the human host cell to obtain growth advantage for themselves intracellularly, while causing multiple changes in the human cell promoting carcinogenesis and cancer spread. The actions of different bacteria may synergize with each other to cause cancer development. With regard to prevention, it will be important to determine the routes of infection of the multiple cancer-associated anaerobic pathogens. Future studies need to be directed towards screening and treatment of specific anaerobic bacterial infections, assessing the value of antimicrobial therapies in preventing cancer progression.

## supplementary material

10.1099/jmm.0.001817 Uncited Table S1.
