# Supplementary material for: Cancer invasion and anaerobic bacteria: new insights into mechanisms
Source: J Med Microbiol. 2024 Mar 28;73(3):001817. doi: 10.1099/jmm.0.001817 (PMC10995961; doi:10.1099/jmm.0.001817)
Supplement: Uncited Table S1. [file jmm-73-01817-s001.pdf]

Supplementary Material for

Cancer Invasion and Anaerobic Bacteria: New Insights into Mechanisms

Rachel Hurst, Daniel S. Brewer, Abraham Gihawi, John Wain, Colin S. Cooper

Journal of Medical Microbiology (2024) DOI: 10.1099/jmm.0.001817

Supplementary file includes:

Supplementary Table 1: Summary of anaerobic bacteria identified in clinical samples reported to be associated with cancer.

Supplementary Table 1: Summary of anaerobic bacteria identified in clinical samples reported to be associated with cancer. Bacteria genera are presented in the order of Bacteria Phyla (*Actinobacteria/Actinomycetota*†, *Bacteroidetes/Bacteroidota*‡, *Firmicutes/Bacillota*∞ and then *Fusobacteria/Fusobacteriota*#), please refer to Table 1 for further details with expanded review of studies/references and association with cancer type.

	Breast cancer	Bladder cancer	Cervical cancer	Colorectal cancer	Endometrial cancer	Gastric cancer	Laryngeal cancer	Lung cancer	Melanoma	Oesophageal cancer	Oral cancer	Pancreatic cancer	Prostate cancer	Acute lymphoblastic leukaemia	Multiple types of cancer	References
Propionimicrobium spp. †													*			[66]
Varibaculum spp. †		*											*			[66,146,147]
Prevotella spp. ‡	*	*	*	*	*	*	*	*	*	*	*				*	[128,130,148-171]
♦***Porphyromonas spp.*** ‡			*	*	*				*	*	*	*	*		*	[10,67,130,137,169-175]
Bacteroides spp. ‡				*		*			*			*	*		*	[76,90,157,162,170,176-179]
♦***Fenollaria/Ezakiella spp.*** ∞		*	*	*	*								*		*	[10,158,165,180]
♦***Peptoniphilus spp.*** ∞	*	*	*		*						*		*		*	[10,76,158,159,165,175,176,181]
♦***Anaerococcus spp.*** ∞		*	*		*								*		*	[10,66,130,131,159,165,182]
Veillonella spp. ∞		*				*		*	*			*		*	*	[128,138,147,163,165,170,179,183]
♦***Fusobacterium spp.*** #	*	*	*	*	*	*	*		*		*	*	*		*	[10,12,13,14,76,128,130,148,150,151,158,159,162,170,178,179,182,184-192]
Sneathia spp. #			*		*										*	[130,131,159]

♦Anaerobic bacteria biomarkers set (ABBS) associated with prostate cancer progression [10] and also associated with several other types of cancer as detailed above.

Hurst *et al.*, Cancer Invasion and Anaerobic Bacteria: New Insights into Mechanisms, Journal of Medical Microbiology (2024), DOI: 10.1099/jmm.0.001817
